# Hippo Signaling in Cancer: Lessons From *Drosophila* Models

**DOI:** 10.3389/fcell.2019.00085

**Published:** 2019-05-24

**Authors:** Kirti Snigdha, Karishma Sanjay Gangwani, Gauri Vijay Lapalikar, Amit Singh, Madhuri Kango-Singh

**Affiliations:** ^1^Department of Biology, University of Dayton, Dayton, OH, United States; ^2^Department of Biochemistry and Biophysics, Texas A&M University, College Station, TX, United States; ^3^Pre-Medical Programs, University of Dayton, Dayton, OH, United States; ^4^Center for Tissue Regeneration and Engineering at Dayton, University of Dayton, Dayton, OH, United States; ^5^Integrated Science and Engineering Center, University of Dayton, Dayton, OH, United States

**Keywords:** *Drosophila*, hippo pathway, cell proliferation, cell death, cell-polarity, cancer

## Abstract

Hippo pathway was initially identified through genetic screens for genes regulating organ size in fruitflies. Recent studies have highlighted the role of Hippo signaling as a key regulator of homeostasis, and in tumorigenesis. Hippo pathway is comprised of genes that act as tumor suppressor genes like *hippo* (*hpo*) and *warts* (*wts*), and oncogenes like *yorkie* (*yki*). YAP and TAZ are two related mammalian homologs of *Drosophila* Yki that act as effectors of the Hippo pathway. Hippo signaling deficiency can cause YAP- or TAZ-dependent oncogene addiction for cancer cells. YAP and TAZ are often activated in human malignant cancers. These transcriptional regulators may initiate tumorigenic changes in solid tumors by inducing cancer stem cells and proliferation, culminating in metastasis and chemo-resistance. Given the complex mechanisms (e.g., of the cancer microenvironment, and the extrinsic and intrinsic cues) that overpower YAP/TAZ inhibition, the molecular roles of the Hippo pathway in tumor growth and progression remain poorly defined. Here we review recent findings from studies in whole animal model organism like *Drosophila* on the role of Hippo signaling regarding its connection to inflammation, tumor microenvironment, and other oncogenic signaling in cancer growth and progression.

## Introduction

Cancer is a complex genetic disease where cells divide uncontrollably and infiltrate normal cells causing debilitating effects often leading to death (Balmain and Akhurst, 2004). Cancer cells activate mechanisms that remove the normal checks on growth and promote tumor growth and survival (Hanahan and Weinberg, 2000, 2011). The current standard of care is surgery, often followed by radiation- or chemo-therapy for treating cancer. However, cancer cells show remarkable abilities to evade immune-surveillance mechanisms and are often resistant to these therapies (Berns and Bernards, 2012). A key question is what are the key cellular events that occur in early stages of cancer? Further, what are the environmental or internal cues that trigger these changes? Although these questions remain unresolved, the vast body of work has revealed the role of cellular signals induced by oncogenic pathways in cancer growth and progression (Zhao et al., 2011). In addition, the focus of such studies is to develop targeted therapies that are more effective and benefit the patients. Specifically, oncogene activation induces signaling outputs that are unique, and cause activation of effectors that promote uncontrolled proliferation of cancer cells (Ohsawa et al., 2014; Enomoto et al., 2015b). One such key effector is the YAP/Yki transcriptional co-activator that acts downstream of the Hippo pathway (Zhao et al., 2008a; Kango-Singh and Singh, 2009; Choi, 2018; Kim and Jho, 2018). In this review, we focus on the insights provided by studies in the *Drosophila* model system, where the pathway was initially identified, on the role of Hippo/Yki signaling in cancer.

## Hippo Signaling in *Drosophila* and Mammals

The regulation of growth through signaling pathways plays a critical role in maintaining tissue homeostasis through the regulation of key cellular processes like cell proliferation and cell death. Signaling pathways comprise of cascade of regulatory proteins that respond to stimulators like growth factors, and influence changes in gene expression that control differentiation, cell migration, cell–cell interaction, immunity, polarity, and metabolism (Davis, 2000; Chen and Wang, 2002; Halder and Johnson, 2011; Bejsovec, 2013; Kim and Jho, 2014, 2018). Disruption of signaling pathways causes an imbalance in the regulation of such mechanisms and leads to diseases such as neuro/muscular degeneration, cancer, diabetes, etc. The Hippo Pathway is a prime example of an important growth regulatory pathway that coordinately controls cell proliferation and survival to regulate organ size (Kango-Singh and Singh, 2009; Kim and Jho, 2018).

**FIGURE 1:**
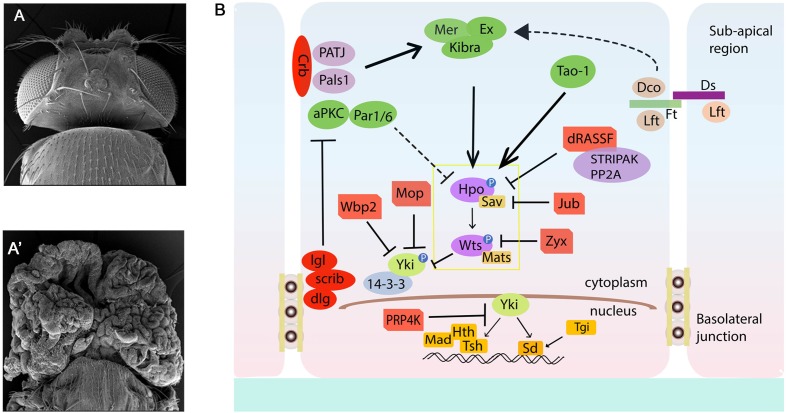
The *Drosophila* Hippo pathway Network. **(A,A′)** Compared to Wild type **(A)**, loss of function of Hippo pathway genes *wts^X1^* (*eyFLP; FRT82B cl w+/FRT82B wts^X1^*) causes significant overgrowth of the *Drosophila* head. **(A,B)** Schematic diagram of the Hippo pathway in *Drosophila*. Cells are shown with Sub-apical region and basolateral junctions. Hippo pathway components in *Drosophila* are shown in different colors, with pointed and blunt arrowheads indicating activating and inhibitory interactions, respectively. Continuous lines indicate known interactions, whereas dashed lines indicate unknown mechanisms. See text for further details. Crb, Crumbs; Dco, Disks overgrown; Dlg, Disks large; Ds, Dachsous; Ex, Expanded; Hth, Homothorax; Jub, Ajuba; Lgl, Lethal giant larvae; Mer, Merlin; Mats, Mob as a tumor suppressor; Rassf, Ras-associated factor; Sav, Salvador; Scrib, Scribble; Sd, Scalloped; TEA domain protein; Tsh, Teashirt; Yki, Yorkie; hpo, Hippo; wts, warts; aPKC, atypical protein kinase C; Wbp2, WW domain binding protein 2; Mop, Myopic; Zyx, Zyxin; STRIPAK, striatin-interacting phosphatase and kinase; Mad, Mothers against Decapentaplegic; Tgi, Tondu-domain containing growth inhibitor; Ft, Fat; Lft, Lowfat.

**FIGURE 2:**
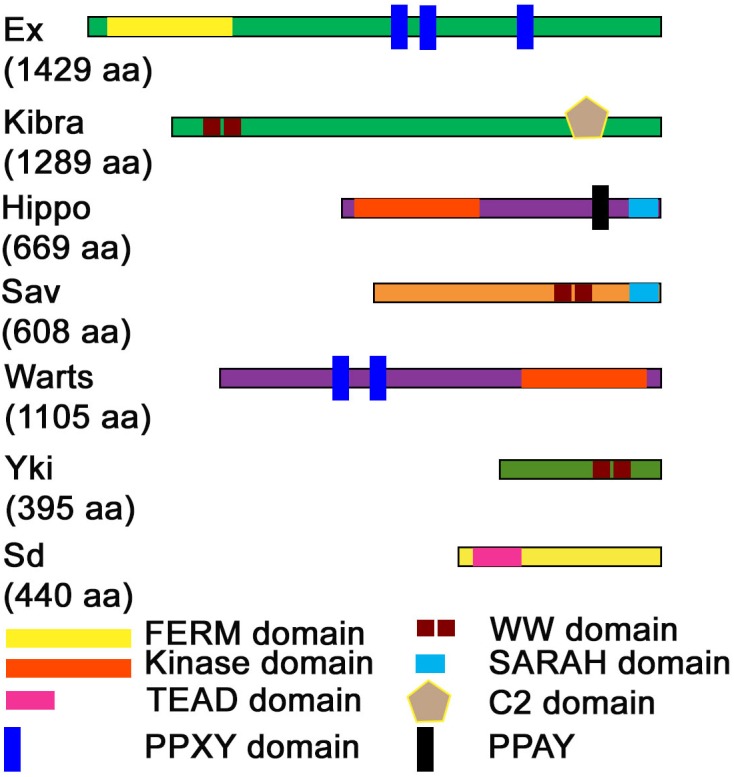
Domain structure of Hippo pathway proteins. The domain structure of Hippo pathway components is shown along with the length of each protein (indicated in parentheses). FERM, Kinase, WW, TEAD, PPXY. PPAY, SARAH, and C2-domains are the prominent domains through which major protein-protein interactions occur in the Hippo pathway.

The Hippo pathway is named after the “big-headed” phenotype [reminiscent of Hippos] of mutants isolated from genetic screens in flies (Figure 1A,A′). Once characterized, these mutations were found to belong to three key genes, *warts* (*wts a.k.a. large tumor suppressors*, *lats*), *salvador* (*sav* a.k.a. *sharpei, shrp*) and *hippo* (*hpo*, a.k.a. *Drosophila mammalian Ste-20 kinase*, *dMst*) (Justice et al., 1995; Xu et al., 1995; Kango-Singh et al., 2002; Tapon et al., 2002; Harvey et al., 2003; Jia et al., 2003; Pantalacci et al., 2003; Udan et al., 2003; Wu et al., 2003). Hpo coded for a Serine Threonine kinase and was orthologous to the Ste-20 kinases that were previously found to play an important role in pheromone sensing and mating in yeast (*S. cerevisiae*) (Creasy and Chernoff, 1995; Dan et al., 2001). Studies further showed that the Hippo kinase functioned with Lats (or Wts) another previously discovered Serine-Threonine kinase in flies which is a Nuclear Dbf2-related (NDR) family kinase (Justice et al., 1995; Xu et al., 1995). Hpo and Wts were also shown to interact with Sav, a WW-domain containing adaptor protein (Kango-Singh et al., 2002; Tapon et al., 2002). Interestingly, loss of *sav*, *hpo*, or *wts* in somatic clones caused tissue overgrowths (Figure 1A,A′) and extra interommatidial cells in the pupal retina. The characterization of these phenotypic defects showed that these genes possess the rare ability to promote proliferation and suppress apoptosis simultaneously (Edgar, 2006). These early discoveries lead to the birth of the Hippo pathway in 2003 as a nascent network that is capable of simultaneously regulating two key processes – cell proliferation and apoptosis, and plays important role in maintaining homeostasis. The Hippo pathway gained tremendous attention when Yorkie (Yki, *Drosophila* homolog of mammalian YAP/TAZ) was identified in a yeast two-hybrid screen for Wts binding protein (Huang et al., 2005). YAP (Yes-Associated Protein) was identified in vertebrates much before Yki was identified in the fruitfly (Sudol, 1994). With the identification of these key components (Figure 1B, 2), it was soon clear that the Hippo pathway is conserved in mammals, plays a key role in development, and is misregulated in many disease conditions.

YAP was initially discovered via its ability to associate with the Src family member Yes, while its paralog transcriptional coactivator with PDZ-binding motif (TAZ a.k.a. WW domain-containing transcription regulator 1, WWTR1) as a novel 14-3-3 binding protein (Sudol, 1994; Kanai et al., 2000), and many seemingly unconnected properties of YAP/TAZ were reported (Figure 2). For example, studies showed that WW domain of YAP can bind directly to proline-rich PPXY motifs in proteins named WBP-1 and WBP-2, and this was the very first molecular function ascribed to YAP (Chen and Sudol, 1995; Sudol et al., 1995; Pirozzi et al., 1997; Wang et al., 2009; Sudol, 2010). Other subsequent studies showed that YAP and TAZ have transcriptional co-activator activity via association with nuclear DNA binding transcription factors like TEAD p73, p53BP-2, SMAD7, ERBB4, PEBP2α, and RUNX2 (Strano et al., 2001; Vassilev et al., 2001; Hong and Guan, 2012; Misra and Irvine, 2018). Yet another report showed that majority of the YAP/TAZ proteins were sequestered in the cytoplasm in complexes with 14-3-3 proteins that directed them for proteasomal degradation (Ma et al., 2018). Nonetheless, YAP/TAZ functions were not properly understood until the paramount discovery of Yki and its different regulatory mechanisms, which had a great impact on the field of Hippo signaling.

Yki overexpression caused overgrowth phenotypes similar to *sav, hpo*, and *wts* loss of function suggesting that Yki is an oncogene. Biochemical and genetic studies in *Drosophila* revealed that Yki is required for normal tissue growth and its activity is inhibited by Wts-mediated phosphorylation (Wu et al., 2003; Dong et al., 2007; Zhao et al., 2008b). Furthermore, the overgrowth phenotypes associated with inactivation of tumor suppressor genes (*sav, hpo*, or *wts*) were diminished by loss of *yki*, suggesting that Yki is key effector of the Hippo pathway (Wu et al., 2003; Dong et al., 2007; Zhao et al., 2008b). The *Drosophila* Yki and its mammalian counterpart YAP are WW-domain proteins that share 45% sequence similarity (Wang et al., 2009; Sudol, 2010; Chan et al., 2011; Salah and Aqeilan, 2011), and are regulated by phosphate dependent and independent mechanisms (Oh and Irvine, 2009; Oh et al., 2009; Meng et al., 2016).

### Regulation of Yki/YAP by Phosphorylation

Yki/YAP are regulated by the action of the kinase cascade comprised of the Hpo and Wts/Lats kinases (Figure 1B, 2), and their cognate adaptor proteins Sav and Mob as a Tumor Suppressor (Mats) (Oh and Irvine, 2009; Chen et al., 2019). The upstream kinase Hpo phosphorylates Sav, and the activated Hpo-Sav complex in turn phosphorylates and activates Mats and the downstream kinase Wts (Udan et al., 2003; Wu et al., 2003; Huang et al., 2005; Lai et al., 2005; Dong et al., 2007; Wei et al., 2007; Zhao et al., 2007; Oka et al., 2008; Avruch et al., 2012). The activated Wts-Mats complex phosphorylates Yki that controls its activity and nuclear availability (Figure 1B, 2) (Edgar, 2006; Wei et al., 2007; Praskova et al., 2008). In *Drosophila*, Wts regulates Yki by phosphorylating it at site Ser168 (Dong et al., 2007; Hao et al., 2008; Oh and Irvine, 2009). This phosphorylation leads to binding of the 14-3-3 proteins which decreases the nuclear localization of Yki (Oh and Irvine, 2008; Zhang J. et al., 2008; Zhao et al., 2008b; Ren et al., 2010). Growth inhibitory proteins like dRASSF1 (Ras-associated domain family) were identified as interactors of Hpo/MST kinases and Sav/hWW45. RASSF1 competes with Sav for its binding to Hpo through its SARAH domain, and impacts Hpo activity (Colombani et al., 2006; Polesello et al., 2006; Donninger et al., 2011). Wts activity is negatively impacted by the actions of the LIM domain containing proteins Ajuba (Jub), Daschous (Dachs) and Zyxin (Zyx), which affect Wts function by relocalization of Wts to the junctions, or by downregulation of Wts or Expanded levels (Das Thakur et al., 2010; Rauskolb et al., 2011; Enomoto et al., 2015a; Gaspar et al., 2015). Similarly, in mammals (Table 1) activation of Hippo or mammalian MST1/2 kinase is dependent on phosphorylation by the TAO family kinases (TAO1-3) at Thr180/183, respectively (Boggiano et al., 2011; Poon et al., 2011). This phosphorylation event is not only essential for increased catalytic activity but also in the case of MST1/2, in the formation of complexes with the adaptor protein WW-domain containing 1 (SAV1), and Mps One Binder kinase activator-like 1A and 1B (MOB1A/B or collectively, MOB1) (Hong and Guan, 2012; Liu et al., 2012). The MST/Sav complex phosphorylates large tumor suppressor 1/2 (LATS1/2). LATS1/2, in turn, binds MOB1/2 and phosphorylates YAP as well as its paralog, TAZ. Phosphorylated forms of Yki/YAP/TAZ associate with 14-3-3 and are sequestered in the cytoplasm. This prevents them from entering the nucleus and interacting with transcription factors (i.e., TEAD family members and others) and regulating downstream gene targets (Meng et al., 2016; Kim and Jho, 2018; Misra and Irvine, 2018; Plouffe et al., 2018). The *Drosophila* Hippo pathway components and their mammalian counterparts with respective conserved domains are summarized in Table 1 (Roh et al., 2002; Wu et al., 2003; Albertson et al., 2004; Katoh and Katoh, 2004; Praskova et al., 2004; Wang et al., 2004; Loo et al., 2005; Callus et al., 2006; Gallagher and Knoblich, 2006; Willecke et al., 2006; Hao et al., 2008; Mao et al., 2009; Richter et al., 2009; Hyman-Walsh et al., 2010; Yu et al., 2010; Boggiano et al., 2011; Hergovich, 2011; Guo et al., 2013; Jansen et al., 2015; Schimizzi and Longmore, 2015; Sen et al., 2015; Jagannathan et al., 2016; Koch et al., 2016; Simon et al., 2017; Ghosh et al., 2018).

**Table 1:** *Drosophila* and Mammalian Hippo pathway components.

*Drosophila*	Mammals	Conserved domains
Hippo	MST1/2	Ste20 Ser/Thr kinase and SARAH domains
Warts	LATS1/2	NDR Ser/Thr kinase domain
Salvador	WW45/SAV1/WWP4	WW and SARAH
Mats	MOBKL1 A and B	Cys_2_-His_2_ zinc-binding site/Mob1/phocein domain
Yorkie	YAP/TAZ	WW/PDZ and TEAD binding domain
Scalloped	TEAD 1-4 transcription factors	TEA/ATTS and Yki/YAP-binding domains
Merlin	NF2	FERM domain
Kibra	WWC1/WWC2	WW and C2 domains
Expanded	FRMD6 or Willin (human expanded)	FERM domain
Tao-1	TAO 1-3	Protein kinase domain
Crumbs	CRB 1-3	EGF like and Laminin G domains, PDZ and FERM binding motifs
Lethal giant larvae	Lethal Giant Larvae 1-2	LGL2 domain
aPKC	aPKCλ, aPKCς	PKC kinase, PB1 and C1 domains
Mop	His domain phosphotyrosine phosphatase (HDPTP)	PTP motifs
dJub	Ajuba, LIM proteins/LIMD1/WTIP	LIM domains
dZyx or Zyx102	Zyxin	C-terminal LIM domains
Fat	Fat 1-4/Fat-j	EGF-like, Laminin G and Cadherin repeat domains
Daschsous	Dchs ½	Cadherin repeat domain
Disk overgrown	CK1δ, CK1𝜀	Ser/Thr kinase domain
Low fat	Lix1, Lix1L	Unknown
Scribble	Scribble	PDZ domains
Disks large	Disks Large	L27/PDZ/SH3 domains
dMnt	Mad	Basic helix-loop-helix-zipper, Sin-interacting domain
Homothorax	MEIS1	HM (Homothorax-Meis) domain and homeodomain
Tea-shirt	TSH3	Zinc finger domain
Pat J	PAT J	Common L27 domain
Par 1/6	PAR1/6	PDZ domains
dRASSF	RASSF1-6	Ras association and SARAH domains
Stardust	PALS-1/Mpp5	L27/PDZ/SH3 domain and guanylate kinase like domain
Tgi	VGLL4	TDU domains
dSTRIPAK PP2A	STRIPAK PP2A	PP2A Ser/Thr phosphatase complex


Recent studies have discovered multiple phosphorylation sites on Yki/YAP. Whilst Ser168/Ser127 sites are the most important for Yki/Yap regulation, other crucial Wts/Lats phosphorylation sites exist. Ser111 and Ser250 are involved in Yki regulation. Mutations in these sites has shown to reduce phosphorylation of Ser168, thus affecting the regulation of Yki (Oh and Irvine, 2008, 2009; Ren et al., 2010). Studies have also found four additional YAP phosphorylation sites that might influence YAP activity (Ser61, Ser109, Ser164, and Ser381) (Zhao et al., 2007, 2008b; Hao et al., 2008). Though mutation of all five sites in the YAP protein shows stronger YAP activation, Ser381 is a key amino acid that plays a critical role in YAP activation (Zhao et al., 2007, 2010).

Interestingly, recently additional phosphorylation sites were discovered in Yki/YAP. These include two sites in Yki (Ser169 and Ser172) and five additional sites in Yap (Thr63, Ser138, Ser281, Ser351, and Ser384). In YAP, Ser384 is phosphorylated by Casein Kinase I (CK I) which recruits ubiquitin ligase to negatively regulate YAP via the phosphodegron DSGXS (Zhao et al., 2007, 2010; Feng and Irvine, 2009; Sopko et al., 2009). Although a similar phosphodegron is not conserved in Yki, Disks overgrown (Dco*, Drosphila* CK1δ/𝜀 ortholog) inhibits Yki that supports an evolutionarily conserved Yki/YAP-CKI regulatory axis, although different mechanisms may be employed between *Drosophila* and mammals. Besides CK I, a nuclear kinase called PRP4K negatively regulates Yki/YAP by phosphorylation of Yki/YAP in the nucleus to prevent its nuclear accumulation (Cho et al., 2018). This phosphorylation occurs on subset of Wts/LATS phosphorylation sites, inhibits binding of Yki/YAP to Sd/TEAD and exports Yki back to the cytoplasm. Yki was recently shown to shuttle between the cytoplasm and nucleus in response to upstream stimuli. Using live multiphoton microscopy to assess Yki localization, it was recently reported that Yki rapidly shuttles between the cytoplasm and nucleus in epithelial organs. In *wts* mutant cells, the downregulation of Hippo signaling affects the rate of nuclear import of Yki. Yki localization is also linked to the cell cycle where Yki remains cytoplasmic during interphase but during mitosis Yki is nuclear and chromatin enriched in Sd-dependent manner (Manning et al., 2018). Together, these interactions control aberrant gene regulation by prolonged accumulation of Yki in the nucleus, and may constitute a fail-safe mechanism for restricting Yki/YAP activity. Yet another regulatory mechanism causes YAP to switch from an oncogene to a tumor suppressor through phosphorylation by the non-receptor tyrosine kinase Abelson murine leukemia viral oncogene (c-Abl) under DNA damage condition. This leads to the association of YAP with the transcription factor p73. P73 is a paralog of p53 involved in DNA damage response. Phosphorylation of YAP by c-Abl reduces its ability to bind TEAD but promotes binding to p73 and promotes activation of pro-apoptotic genes (Gong et al., 1999; Tsai and Yuan, 2003; Levy et al., 2008; Keshet et al., 2015). However, phosphorylation of Yki is not an absolute determinant of its localization or stability, as phosphorylation independent regulation of Yki is also reported.

### Phosphorylation Independent Regulation of Yki/YAP

These interactions occur via the association of the WW-domains and the PPxY motifs found in several components of the Hippo signaling pathway (Sudol and Harvey, 2010; Salah and Aqeilan, 2011). These interactions occur at several points within the signaling cascade (Figure 1B, 2). Within the kinase cascade, both Hpo [Mst1/2] and Wts [Lats1/2] contain PPxY motif whereas Sav/hWW45 is a WW-domain containing protein. In terms of the pathways effectors, Yki/YAP [TAZ] are WW-domain containing proteins, and WW domains are important for the transcriptional coactivation function of Yki/YAP. Amongst the upstream regulators, Kibra and Itch are WW-domain containing proteins, whereas the PPxY motif is found in flies and mammalian forms of Expanded, Myopic, Dachsous, Fat, Crumbs, and WBP2. Other PPxY containing proteins that interact to regulate the Hippo pathway include Angiomotin, Angiomotin-Like, p73, ASPP1/2, ERB-B4, SMAD1, RUNX, and DVL2 (Salah and Aqeilan, 2011). The WW-domain of YAP also interacts with the PPPY motif of the p73 (Strano et al., 2005). In *Drosophila*, direct interaction of Yki via its WW-domains with the PPxY motif of Ex, Wts and Hpo regulate pathway activity by sequestering Yki in protein complexes by the apical membrane (Badouel and McNeill, 2009; Badouel et al., 2009; Oh et al., 2009). In phosphorylation-independent regulation of Yki, overexpression of PPxY motif of Ex and Wts suppressed Yki mediated transcriptional activation regardless of mutation of the Wts phosphorylation site on Yki (Oh and Irvine, 2009; Oh et al., 2009; Ren et al., 2010). Wpb2 and Myopic (Mop) are two other proteins that contain the PPxY motif which interact with the WW-domains on Yki and aid in regulation of the Hippo pathway (Gilbert et al., 2011; Zhang et al., 2011). Mop, when overexpressed, regulates Yki activity by tethering it to the cytoplasm. Thus, the WW-domain-PPxY motif interactions are used frequently by the constituents of the Hippo pathway, thus playing an important role in its regulation. The unusually high number of WW- and PPxY-motif containing proteins in the Hippo pathway (Figure 2) suggests a modular and iterative mechanism of regulation of pathway activity (Salah and Aqeilan, 2011).

### Yki/YAP/TAZ Function With DNA-Binding Proteins

Yki/YAP/TAZ are transcriptional co-activator proteins without their own DNA binding domain. Thus, Yki/YAP/TAZ works in conjunction with other DNA-binding transcription factors (Figure 1B). Multiple lines of evidence revealed that Scalloped (Sd)/TEAD, is the major binding partner of Yki/YAP/TAZ in regulating gene expression and tissue growth (Goulev et al., 2008; Wu et al., 2008; Zhang L. et al., 2008). A few other DNA partners have been discovered for Yki/YAP/TAZ. For example, in *Drosophila* Yki can bind Homothorax (Hth) which works in a complex with two other DNA-binding proteins called the Extradenticle and Teashirt (Alarcon et al., 2009; Peng et al., 2009; Oh and Irvine, 2011; Rauskolb et al., 2011). These interactions regulate transcription of target genes that regulate cell cycle progression (e.g., cyclin E, A, B, and D), cell survival [*Drosophila* inhibitor of apoptosis protein 1 (*Diap1, Survivin, Xiap1*)], and cytoskeletal architecture (F-actin, Merlin, Expanded). Yki/YAP/TAZ can interact with other TGFβ signaling pathway DNA binding factors like Mothers against Dpp (Mad) in *Drosophila*, or SMAD1-4 and 7 in mammals (Padgett et al., 1998; Ferrigno et al., 2002; Varelas et al., 2008; Alarcon et al., 2009). The interaction of these binding factors with Yki/YAP/TAZ influences cell proliferation, development, and homeostasis.

### Sd/Yki Dependent Events

Normally the Yki/Sd complexes induce expression of target genes (ex, diap1, etc.), however, the *Drosophila* E2F1 impedes the binding of Yki to Sd, which results in release of Yki from Sd and suppression of target gene expression (Zhang et al., 2017). These studies provided new insights on how Yki/Sd dependent functions can be impacted by the action of other pathways like Rb/E2F that influence formation of the Yki/Sd complexes (Zhang et al., 2017). The emergence of these non-canonical inputs into Hippo signaling along with the complexities of transcriptional regulation of Yki target genes by more than one pathway (e.g., regulation of diap1 by JNK, Yki, and JAK-STAT pathways) lead to the development of genetic strategies/tools that will allow confirmation of Yki/Sd dependent events using epistasis based approaches (Yu and Pan, 2018).

### Hippo Pathway in Mechanotransduction

The Hippo pathway plays a role in mechanotransduction, as Yki activity is affected in response to the mechanical stretch forces in both mammals and flies (Fletcher et al., 2018). In mammals, mechanical forces acting via Integrin adhesion and actin cytoskeleton can induce YAP/TAZ nuclear translocation and activation of target genes leading to cell proliferation. Although Integrins do not seem to be involved in mechanotransduction and Yki mediated induction of cell proliferation in flies, other mechanisms that respond to biomechanical stretch forces have been identified. For example, the tension-dependent recruitment of Ajuba family proteins by a-catenin to the adherens junctions affect Yki activity by inactivation of the Wts kinase. Thus, a-catenin acts as a mechanotransducer that affects regulation of Yki activity (Alegot et al., 2019). Mechanical forces can also activate Yki in response to stretching of the apical domain which affects concentration of Crumbs, Expanded, Merlin and Kibra, and reduced the apical Hippo kinase dimerization (Fletcher et al., 2018). Recently, transcription independent function of Yki was shown where Yki accumulation at the cell cortex in the apical junctional regions promotes activation of myosin through Stretchin-Mlck, a myosin regulatory light chain kinase (Xu et al., 2018).

## Upstream Regulators of Hippo Pathway

Several other components that influence cell contact/junction, cell polarity, and stress induced response were identified as regulators of the Hippo pathway from the receptor to the nucleus in a cell intrinsic manner (Figure 1B, 2).

### Cell Contact/Junction

These comprise the FERM- (4.1, Ezrin, Radixin, and Moesin) domain proteins Merlin (Mer) and Expanded (Ex), the WW- and C2-domain containing protein Kibra that form a complex in the subapical region and respond to signals from the apical transmembrane protein Crumbs (Crb) (Figure 1B, 2) (Hamaratoglu et al., 2006). Loss of function of these genes caused overgrowth phenotypes in somatic clones, suggesting that these upstream components acted as activators of the Hippo pathway. Mer and Ex associate with each other, and are partially redundant genes. Simultaneous loss of *mer* and *ex* show defects that resemble effects of loss of *wts* or *hpo*, or overexpression of Yki; and activate Yki and its transcriptional targets (Hamaratoglu et al., 2006, 2009; Chen et al., 2010). Overexpression of mammalian Merlin homolog, Neurofibromatosis Type 2 (NF2) can activate Lats and cause inhibition of YAP activity (Zhao et al., 2007; Zhang et al., 2009). A third component called Kibra, associates with Mer and Ex, and co-localizes to form a complex that aids in the phosphorylation of Wts and Hpo (Baumgartner et al., 2010; Genevet et al., 2010; Yu et al., 2010). These three members form protein complexes that influence pathway activity. For example, Mer and Kibra can bind to Sav, whilst Ex binds to Hpo, and Kibra binds to Wts (Baumgartner et al., 2010; Genevet et al., 2010; Yu et al., 2010). In addition to binding to the Hippo kinase cascade, Kibra can also bind to Yki, thus keeping Yki in the cytoplasm (Badouel and McNeill, 2009; Badouel et al., 2009; Oh et al., 2009). *Drosophila* Crumbs (Crb), a cell surface regulator for the Hippo pathway, has an intracellular FERM-binding domain that binds to Ex and controls its stability and localization, thus impacting its activity on Hpo kinases and Yki (Chen et al., 2010; Grzeschik et al., 2010; Ling et al., 2010; Robinson et al., 2010; Varelas et al., 2010). A second group of cell-junction related upstream regulators of the Hippo pathway is comprised of the proto-cadherin Fat (Ft) and its interacting components like Dco and Lowfat that negatively regulate the atypical Myosin Dachs and Zyxin to negatively regulate Wts (Bennett and Harvey, 2006; Cho et al., 2006; Silva et al., 2006; Willecke et al., 2006; Baumgartner et al., 2010; Chen et al., 2010; Rauskolb et al., 2011; Matakatsu and Blair, 2012; Verghese et al., 2012a; Choi, 2018). This non-canonical branch of the Hippo pathway ultimately influences the regulation of Yki by the Wts kinase (Cho et al., 2006; Choi, 2018). Together, these components provided the framework for signal transduction from the membrane to the kinase cascade within the cell, a key intracellular signal relay mechanism in Hippo pathway.

### Cell Polarity

Cell polarity regulating complexes have emerged as major upstream interactors of Hippo pathway that control growth by regulating Yki activity (Figure 1B) (Verghese et al., 2012a). Overexpression of Atypical Protein Kinase C (aPKC), which belongs to the Par apical complex, can induce Yki activity and tissue proliferation (Grzeschik et al., 2010; Parsons et al., 2010; Sun and Irvine, 2011). One mechanism is the activation of Jun N-terminal kinase (JNK) which in conjunction with aPKC regulates Yki activity in a context dependent manner (Karpowicz et al., 2010; Staley and Irvine, 2010; Sun and Irvine, 2011). Another complex known as the Scribble (Scrib) complex is antagonistic to the Par apical complex (Leong et al., 2009; Skouloudaki et al., 2009; Verghese et al., 2012a). Reduction in *scrib* leads to loss of polarity and cell adhesion, and causes tumor growth in part due to activation of Yki (Verghese et al., 2012a; Waghmare and Kango-Singh, 2016). Although some gaps remain in how exactly each of these inputs feeds into the core kinase cascade (Figure 1B), the identification of these interactions lead to recognition of the Hippo network and how different inputs are integrated into the Hippo pathway.

### Hippo Pathway and Apoptosis

Hippo overexpression was shown to induce apoptosis by activation of the proapoptotic gene *head involution defective* *(hid)* (Udan et al., 2003). Work from our lab showed that Hpo gain of function induces Dronc (*Drosophila* Caspase-9 homolog) expression and downregulation of *dronc* can inactivate Hpo-mediated apoptosis (Verghese et al., 2012b). Key regulators of apoptosis include p53 (*Dmp53* in *Drosophila melanogaster*) and its homologs P63 and P73 that comprise members of the p53 family of tumor suppressor genes. In *Drosophila*, p53 is activated in response to DNA damage or other stress, and induces transcription of proapoptotic genes ultimately causing cell death. Hippo pathway is activated in response to stress induced by ionizing radiation in a Dmp53 dependent manner (Colombani et al., 2006). Further, IR mediated cell death is reduced in cells mutant for *hpo*, *wts*, *sav*, or *Dmp53.* Furthermore, pro-apoptotic gene *reaper (rpr)* is regulated by Yki and *dmp53*; and is shown to trigger apoptosis via activation of the Hippo pathway (Shlevkov and Morata, 2012). Together these studies showed that Hpo activation is required for cell death in response to IR or ectopic expression of Dmp53. In other studies, LATS mediated phosphorylation of ASPP1 was shown to cause association of ASPP1 and p53, and promote expression of pro-apoptotic genes like *reaper*, *hid, grim*, and *sickle* (Zhang and Cohen, 2013). Another mechanism by which Hippo pathway affects apoptosis is via miRNA mediated translational control of apoptosis causing genes. For instance, Yki regulates miR2 family miRNAs to regulate the translation of *reaper* (Thermann and Hentze, 2007), or the *bantam* miRNA to control translation of *hid* (Brennecke et al., 2003; Nolo et al., 2006; Thompson and Cohen, 2006). Anoikis is a special type of cell death induced due to detachment of cells from the extracellular matrix (ECM). Resistance to anoikis is a hallmark of cancer. Cell detachment activates the Hippo pathway kinases Lats1/2 and leads to YAP phosphorylation and inhibition. In contrast, in cancer cells with deregulated Hippo pathway, anoikis is restored by knockdown of YAP/TAZ (Zhao et al., 2012). Overall, the Hippo pathway plays a role in regulating developmental apoptosis, anoikis, and other cellular interactions in response to stress (e.g., cell competition) that ultimately trigger cell death.

## Hippo Pathway and *Drosophila* Cancer Models

**FIGURE 3:**
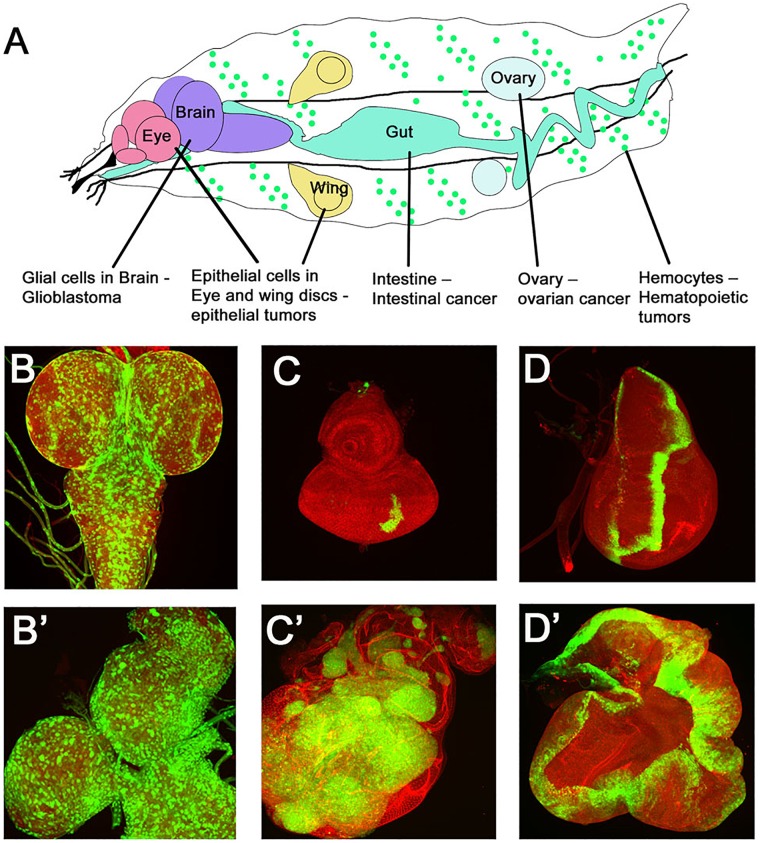
*Drosophila* cancer models. **(A)** Cartoon showing various larval organs/cells in which *Drosophila* cancer models have been developed. The many tissue specific manipulations possible in *Drosophila* have generated tissue specific tumor models like epithelial tumors, glioma, hematopoietic tumors, intestinal tumors, and germ-cell tumors. Examples of some models are shown in **(B–D)**. **(B,B′)** show confocal images of GFP marked glial cells in normal versus a glioma brain model. Note the enlarged brain lobes and increased number of GFP expressing glial cells. **(C–D′)** Epithelial tumors modeled in imaginal disks are shown. Panels show a comparison of a normal eye disk **(C)** with a disk carrying GFP labeled tumors **(C′)** caused by overexpression of oncogenic Ras in polarity deficient *scribble* mutant cells (*Ras^V 12^, scrib^-/-^*) induced by MARCM technique. **(D,D′)** Wild type wing imaginal disk showing a stripe of GFP expression in the *patched-GAL4* domain (*ptcGAL4>UASGFP*) is compared to overgrowths caused by overexpression of the activated form of Hippo pathway effector Yki (*ptcGAL4>UASGFP, UASYki^3SA^*).

***Drosophila*** with its rich repository of mutant genes has played a central role in identifying genes underlying cancer, e.g., ***lethal(2) giant larva (lgl)*,** and ***dis****k****s-large (dlg)*** represent the earliest polarity mutants showed dramatic effects on growth, invasion and malignant metastasis (Gateff and Schneiderman, 1969**;** Bryant and Schubiger, 1971**;** Gateff, 1978). Since then, a large array of oncogenes and tumor suppressor genes have been identified in ***Drosophila***. In addition, the powerful genetic toolkit in ***Drosophila*** has played a central role in developing techniques that allow generation of clonal patches of tumors by loss- or gain-of-function of tumor suppressor genes or oncogenes, or using the Mosaic Analysis with Repressible Cell Marker (MARCM) technique which allows for expression of genes in somatic clones marked positively by reporter genes like GFP (Lee and Luo, 2001). The MARCM technique allows for simultaneous manipulation of two or more genes to generate complex genotypes. For example, mosaic clones can be induced for loss of function of a tumor suppressor genes and in the same cells an oncogene or cell cycle regulator can be overexpressed (Waghmare and Kango-Singh, 2016). These clonal tumors in developing ***Drosophila*** tissues like imaginal disks, gut, muscles, blood cells, ovaries, or CNS can be studied for changes in cell–cell interactions, signaling and gene expression, and the identification of inhibitory drugs (Figure 3).

## Oncogenic Cooperation-Models for Cell–Cell Interactions, Tumor Signaling, and Altered Gene Expression

The first mosaic cancer models in *Drosophila* were reported when activation of oncogenic Ras or Notch was combined with loss of polarity mutants in mosaic clones in imaginal disks (Brumby and Richardson, 2003). These *Ras^V 12^ scrib-* or *N^Act^* *scrib-* MARCM clones showed remarkable properties like increased proliferation, reduced apoptosis, and key changes linked to tumor invasion and metastasis. Using similar approaches, a genetic mosaic screen was conducted to identify metastasis causing genes in flies (Pagliarini and Xu, 2003). These studies identified second site mutations that cooperated with activated Ras to cause tumorigenesis. The development of these epithelial tumor models opened up the field for studying the step-wise progression of cancer especially the initial changes underlying the transformation of cells, and the resulting changes in cell behavior and gene expression. Over the last 15 years the oncogenic cooperation models (*Ras^V 12^ scrib^-^*) have resulted in an improved understanding of the addiction to Yki activation in cancer cells (Ma et al., 2018), the interaction of Yki with other signaling pathways (Zecca and Struhl, 2010; Sun and Irvine, 2013; Enomoto et al., 2015a; Piersma et al., 2015), and ectopic activation of network of transcription factors (Kulshammer et al., 2015; Atkins et al., 2016).

### Yki Addiction in Cancer Cells

The epithelial tumor models of oncogenic cooperation which involved activation of oncogenes in polarity deficient cells (e.g., *Ras^V 12^ scrib^-^*) result in increased Yki activity which is required for aggressive tumor growth (Brumby and Richardson, 2003; Uhlirova et al., 2005; Suijkerbuijk et al., 2016). Similarly, models of intestinal adenomas (APC^-/-^ cells) show increased Yki activity that promotes tissue growth (Hall et al., 2010; Chen L. et al., 2012; Chen C.L. et al., 2012; Suijkerbuijk et al., 2016). *Drosophila* intestinal stem cell tumors (ISC) induced by Notch suppression cause displacement of enterocytes from the epithelium and induce increased Yki activity which promotes tumor growth (Patel et al., 2015). Other cancer models in several *Drosophila* tissues or cell types also revealed a tendency to elevate Yki expression and activity. Examples of such models include intestinal malignancies (Chen L. et al., 2012; Meng et al., 2016), melanotic hematopoietic tumors caused by activation of Hopscotch (Hop) the *Drosophila* Janus Kinase (JAK) (Anderson et al., 2017), and ovarian cancer (Hall et al., 2010). Recently, Yki was shown to cooperate with microRNA *mir8* and cause neoplastic tumors (Sander et al., 2018). In a separate study, cell competition, a key cell–cell interaction mechanism that compares relative fitness of cells was shown to act as a tumor suppressor mechanism that regulates Yki activity. Cells where relative Yki activity levels are low (e.g., *scrib^-/-^* cells) are eliminated by competition mediated apoptosis, however, elevation of Yki activity is sufficient to induce neoplastic growth in such cells (e.g., *Yki, scrib^-/-^* cells) (Chen C.L. et al., 2012). Using *Drosophila* cancer models the high nutritional demands of activated Ras/Src cancer cells revealed interesting links between high sugar diet (obesity), cancer growth and downregulation of Hippo pathway (Hirabayashi et al., 2013; Hirabayashi and Cagan, 2015). This study revealed that Ras/Src transformed cells are sensitive to upstream Hippo signals, and that Yki dependent signaling through activation of the Salt Inducible Kinase (SIK) is a key feed-forward mechanism for evasion of insulin resistance and tumor growth in diet-induced obesity and cancer.

A large body of work in mammalian models has provided evidences for YAP/TAZ addiction in cancer cells, and increased YAP activity and expression is associated with advanced stages of cancer progression and poor prognosis. Several human cancer cells show increased nuclear translocation of the YAP protein indicating the suppression of the Hippo pathway. Similarly, in breast cancer patient samples, elevated expression of YAP/TAZ has been found and associated with the poor prognosis, stem cell and metastasis. Multiple Ankyrin repeats Single KH domain protein (Mask) was shown to be elevated in breast cancer samples, and MASK promoted Yki expression and is required for full activity of YAP/TEAD. Overexpression of YAP was sufficient to transform normal ovarian cancer cells and induce tumorigenesis in athymic nude mice (Li et al., 2017). These studies found that Hippo pathway and ERBB signaling pathway to drive the ovarian tumor initiation and progression. In patient samples and in preclinical *Drosophila* models, Arrestin-related domain-containing protein-3 (ARRDC3) was downregulated in the colorectal cancer specimens. ARRDC3 promoted YAP degradation, increased drug sensitivity of the tumor cells and was proposed as a potential drug target (Shen et al., 2018). In another study, loss of Mst1/2 or conditional overexpression of YAP in response to bile acid induced injury in liver cells was shown to cause Hepatocellular carcinoma (HCC) in mice models. However, recent studies also show that *yap/taz* mutant mice can also develop liver adenomas possibly by creating an environment that mimics chronic liver injury. Liver damage and associated conditions like liver fibrosis and cirrhosis is known to create tumor-promoting microenvironment formed by chronic inflammation, and leads to activation of Hippo/YAP and other signaling pathways, ultimately causing hepatocellular carcinoma (Piersma et al., 2015; Kodama et al., 2018; Lu et al., 2018). Evidence supports the role of Hippo signaling transcriptional co-activator TAZ in promoting Glioma, a primary brain tumor, through transition of glioma stem cells (GSCs) to the Mesenchymal type (MES) (Bhat et al., 2011; Waghmare et al., 2014). These transitions are marked by transition from proneural (PN) gene expression profile to mesenchymal (MES) – a signature linked to glioma recurrence and therapy resistance. Recently, the glycoprotein CD109 was shown to associate with many cancers including GBM; and CD109 and YAP/TAZ are known to regulate some overlapping biological pathways in cancer. Using mammalian and *Drosophila* glioma models it was shown that CD109 (Tep 1 in *Drosophila*) regulates YAP/TAZ transcriptional activity via a conserved pathway. These studies propose that the conserved regulation of YAP/TAZ pathways by CD109 could be a therapeutic target in GBM (Minata et al., 2019). In summary, increased Yki/YAP activity resulting from feedback loops or overexpression of Yki/YAP is frequently associated with aggressive tumorigenesis. Further, both in flies and mammalian models, addiction to Yki is a key property of cancer cells.

## Interaction of Yki With Other Signaling Pathways During Tumor Progression

*Drosophila* cancer models revealed that a complex cross-talk between Hippo effector Yki and other signaling pathways is required for tumor growth and progression. In the oncogenic cooperation model where activated Ras oncogene is expressed in polarity deficient cells (*Ras^V 12^; scrib****^-^***), the Ras-MAPK pathway is activated downstream of Ras (Doggett et al., 2011; Enomoto et al., 2015b). In addition, several signaling pathways are induced during tumorigenesis (Sanchez-Vega et al., 2018). Studies in the *Ras^V 12^; scrib****^-^*** metastasis model have shown interaction with EGFR, JNK, Wingless, JAK-STAT, TNF, TGFb, microRNAs, GPCRs (G-proteins), which are briefly described in the sections below.

### Hippo and EGFR-Ras-Raf-MAPK Pathway

Activated Ras (RasV12/ or other KRAS/BRAF mutations) is found in one-fifth of all cancers, and is associated with activation and amplification in EGFR and its downstream effectors. Studies in *Drosophila* and other model systems showed that hyperactivation of Ras causes only mild over-proliferation or hyperplasia. However, oncogenic Ras when co-occurring with other mutations like *p53*, loss of cell polarity proteins [*scrib^-^* and *dlg^-^*], or components of the JNK and Hedgehog (Hh) signaling pathways caused aggressive metastatic tumors (Scheffler et al., 2018). Using *Drosophila* mosaic models, it was shown that Hippo switches Ras activation from promoting cellular differentiation to aggressive cellular proliferation by directly controlling two Ras pathway genes- the transcription factor Pointed (Pnt) and the repressor protein Capicua (Cic) (Pascual et al., 2017). Loss of function of Cic in flies and mammals (e.g., in Oligodendroglioma) phenocopies activation of Ras/MAPK pathway, and uncontrolled aggressive proliferation (Weissmann et al., 2018).

### Hippo and JNK Pathway

The Jun N-terminal Kinase (JNK) is a MAPK pathway regulated by many cellular stressors like disruption of cell polarity, DNA damage caused by radiation or reactive oxygen species, and activation of apoptosis etc. JNK pathway elicits context dependent response that commonly toggles between proapoptotic or pro-proliferation signaling. For example, in the context of wound healing, increased JNK activity pushes cells into apoptosis, however, when apoptosis is blocked JNK activity switches to a pro-proliferation signal. Furthermore, JNK signaling is activated in metastatic tumors formed by polarity deficient cells (Igaki et al., 2006; Uhlirova and Bohmann, 2006). The earliest evidence for a connection between Hippo pathway and the Jun N-terminal Kinase (JNK) was noted when JNK was shown to mediate activation of Yki in polarity deficient neoplastic cells or when pro-apoptotic genes were expressed. This activation of Yki by JNK was thought to be a part of regenerative response to tissue damage (Karpowicz et al., 2010). In a separate study, Yki activation in endocytosis-defective cells is accompanied by activation of the JNK signaling pathway, a MAP kinase pathway that has been linked to *Drosophila* neoplasia and control of Yki activity (Robinson and Moberg, 2011). Overall, these data illustrate the paradoxical nature of JNK signaling and its many context-dependent interactions with Yki/YAP.

### Hippo and Notch Pathway Crosstalk

The Hippo pathway interact with Notch in many cell types suggesting that the Notch/Yki cross talk has important regulatory roles in growth control, differentiation and in other contexts (Reddy et al., 2010; Graves et al., 2012; Ferguson and Martinez-Agosto, 2014). In the developing optic lobe of *Drosophila* larval brain, the neuroepithelial cells are sheets of epithelial neural progenitor cells that function as symmetrically dividing neural stem cells. Before converting to neuroblasts (neural stem cells), the neuroepithelial cells undergo cell cycle arrest that is regulated by the Hippo pathway. Neuroepithelial cells mutant for pathway genes like *wts* or overexpression of activated Yki result in overproliferation of the neuroepithelial cells and delays differentiation. The progression of neuroepithelial cells to neuroblasts is regulated by Notch signaling, where high levels of the Notch ligand Delta inhibits Notch activation and promotes neuroblast fate. The Hippo pathway impairs Delta accumulation and thus affects differentiation of neuroblasts. These studies showed that cross talk between Yki/Sd and Notch pathway plays a key role in neuroepithelial growth and differentiation (Reddy et al., 2010). The *Drosophila* endocytic neoplastic tumor suppressor genes like Vps25 is part of the Endosomal Sorting Complex Required for Transport-II (ESCRT-II) complex. In the epithelial imaginal disk cells, loss of *vps25* causes sorting defects and activation of Notch, JAK-STAT and JNK signaling pathways, however, mutant clones of *vps25* are unable to survive and are eliminated (Thompson et al., 2005; Herz et al., 2006). Interestingly, before dying *vps25* mutant clones induce Notch signaling which, in turn, non-cell autonomously induces Yki activation that results in tissue overgrowth (Graves et al., 2012). Notch and Yki/Sd interactions are also known for the specification of crystal cells during hematopoiesis (Ferguson and Martinez-Agosto, 2014). Yki and Sd regulate expression of Serrate, the ligand for Notch, which is responsible for triggering the differentiation program for crystal cells. These studies show a role of Yki/Sd in progenitor cell niches that are required for specifying cell fate (Ferguson and Martinez-Agosto, 2014).

### Intersection of TOR and Hippo Pathway

Hippo and TOR pathways intersect in diverse contexts during development and in autophagy in *Drosophila*. Cell growth arrest and autophagy are important for metapmorphosis, and the autophagic cell death of the salivary glands is an important developmental process. Wts the terminal kinase in the Hippo pathway was shown to be required for salivary gland degradation. Further, Wts mediated salivary gland cell death was shown to be dependent on PI3K pathway (Dutta and Baehrecke, 2008). In a different study in human Hepatocellular carcinoma as well as in *Drosophila* imaginal disks, PI3K signaling was shown to affect Yki activity and *vice-versa* (Strassburger et al., 2012). The nutrient dependent systemic signaling through the TOR pathway is important for organ growth and in maintenance of adult homeostasis in flies and mammals (Wullschleger et al., 2006; Neto-Silva et al., 2009). Using *Drosophila* wing imaginal disks as a model for growth regulation, Parker and Struhl (2015) showed that TOR regulates Yki via an interesting mechanism referred to as the ‘seclusion mechanism.’ In this mechanism, inhibition of TOR leads to Yki accumulation in the nucleus, however, Yki does not regulate transcription as TOR inhibition impedes access to target genes of both Yki and Sd. Thus, TOR promotes wing growth in response to two parallel pathways, one nutrient availability, and second by controlling Yki activity (Parker and Struhl, 2015). Mammalian studies have also shown interactions between YAP and mTOR. Recently, it was shown YAP downregulates PTEN by inducing miR-29 to inhibit PTEN translation. PI(3)K-mTOR is a pathway modulated by YAP to regulate cell size, tissue growth and hyperplasia (Tumaneng et al., 2012).

### Ectopic Activation of Transcription Factor Networks in Cancer Cells

The different models of oncogenic cooperation demonstrated the range of signaling interactions involved in the tumorigenic process. Genetic, biochemical, and high-throughput RNAseq approaches have revealed the spectacular diversity in altered cellular signaling often via interacting transcription factors. These aberrant interactions are central to promoting tumorigenesis. In the oncogenic cooperation model of epithelial *Ras^V 12^ scrib^-^* tumors, the interactions between JNK and JAK-STAT pathways was detected as activation of JNK caused induction of Unpaired 3 (Upd3, *Drosophila* IL-6 like cytokine) resulting in systemic induction of JAK-STAT signaling that promotes tumor growth (Brumby and Richardson, 2003; Igaki et al., 2006; Pastor-Pareja et al., 2008; Wu et al., 2010). Consistent with the idea of STAT playing a key role in tumorigenesis, the cooperation of oncogenic Ras with activated Stat92E (*Ras^V 12^; Stat92E*) was shown to be sufficient to cause tumor growth and invasion (Wu et al., 2010). JAK-STAT misregulation was shown to be sufficient for melanotic and hematopoietic tumors in *Drosophila*, however, many other instances where modifiers of JAK-STAT or JNK and JAK-STAT interactions were reported. For example, in tumors caused by cooperative interactions between activated Ras and mitochondrial defects (Ohsawa et al., 2012) and intestinal tumors (Kolahgar et al., 2011; Suijkerbuijk et al., 2016). Other research on epithelial tumors revealed an intricate interaction between JNK and Yki which was traced to a polarity-responsive enhancer in Upd3 activated by JNK-dependent Fos and aPKC-mediated Yki transcription. Using unbiased approaches transcription factors of several families that act downstream of JNK were identified. These include bZIP protein Fos, the ETS-domain factor Ets21c and the nuclear receptor Ftz-Fdem1. Furthermore, although all three transcription factors were required for aggressive tumorigenesis, the synergistic requirement of a subset of transcription factors for invasiveness and tumor growth generated deeper insights on the unique and overlapping functions of transcription factors that cooperatively activate an array of tumor promoting target genes (Kulshammer et al., 2015). Recently a JNK-dependent AP1 responsive enhancer was mapped in Wingless, and interaction between JNK and Wingless promoted growth of epithelial tumors (Zhang et al., 2019). Yki/Sd mediated transcriptional interactions have also been investigated in multiple tumor models. For example, Yki/Sd interact with Src and JNK (Enomoto and Igaki, 2013); and with Stat, AP-1, Myc and AP-4, Ftz-f1, Taiman/SRC3, and Mef2 (Atkins et al., 2016) to promote tumorigenesis. Many of these transcriptional interactions are also seen in mammalian cancers suggesting that some transcriptional networks are evolutionarily conserved. Taken together, these studies showed that the Hippo pathway (particularly Yki/YAP) interacts with multiple signaling pathways to maintain homeostasis, however, complex and unique tumor specific interaction networks are formed during tumorigenesis where Yki/YAP interact with other signaling pathways only in tumor cells.

## Future Directions and Concluding Remarks

There are additional Ste-20 kinases possibly belonging to the NDR family found in mice which might indicate that these kinases might also have a role in phosphorylation and growth control (Li et al., 2014; Meng et al., 2015; Zheng et al., 2015). These kinases are thought to influence phosphorylation of Ser127, a major regulatory site of YAP. These understudied kinases and their *Drosophila* orthologs need to be further characterized to define their cell-type and context specific roles to better define the Hippo pathway. Further, if Wts is the sole regulator of Yki or other NDR family components act in parallel to Wts/Lats to control Yki/YAP, and if these pathways cross-talk in specific contexts are interesting aspects of the Hippo pathway function and regulation that currently are not clearly defined. Each year new components are added to the Hippo network, for example, recently Schip1 was shown to connect Expanded to the Tao-1 kinase (Chung et al., 2016). However, if Schip1 interacts with other upstream components or other inputs that influence Yki activity needs further investigation. In addition, other genes that act upstream of the Kibra/Merlin/Expanded complex and the Tao1 kinase remain to be identified. Thus, in the future it will be interesting to find the missing links within the Hippo pathway, the mechanisms underlying their interactions with the core pathway machinery and whether it affects tumorigenesis.

## Author Contributions

KS, KG, GL, AS, and MK-S discussed review outline. KS, KG, and GL performed literature search and created figures. KS, KG, GL, and AS wrote the initial drafts. MK-S wrote and edited the final manuscript.

## Conflict of Interest Statement

The authors declare that the research was conducted in the absence of any commercial or financial relationships that could be construed as a potential conflict of interest.
